# Linking smoking with pulmonary function among COPD patients in India

**DOI:** 10.6026/973206300213350

**Published:** 2025-09-30

**Authors:** Darshi Rastogi, Pankaj Kumar Gupta, Ronak Kumar N. Patel, Rahul Soni, Sachin Parmar

**Affiliations:** 1 Department of Pulmonary Medicine, LN Medical college & JK Hospital, Bhopal, Madhya Pradesh, India; 2 Deprtmentof General Medicine, BMGMC Shahdol, Madhya Pradesh, India; 3 Deprtmentof Respiratory Medicine, BMGMC Shahdol, Madhya Pradesh, India; 4 Department of Community Medicine, V.K.S. Government Medical College, Neemuch, Madhya Pradesh, India

**Keywords:** Chronic Obstructive Pulmonary Disease (COPD), smoking, spirometry, pulmonary function, FEV_1_, FVC, India, chronic obstructive pulmonary disease

## Abstract

The correlation between smoking history and pulmonary function among COPD patients in Central India is of interest. Data from 206 COPD patients were analyzed to examine the impact of smoking on lung function, using spirometry measurements of FEV_1_ and FVC. Data shows a significant negative correlation between smoking history (duration and smoking index) and lung function, particularly in FEV_1_ and FEV_1_/FVC ratios. Thus, we show the critical role of smoking in exacerbating COPD severity and highlight the importance of early intervention and smoking cessation to improve patient outcomes.

## Background:

Chronic Obstructive Pulmonary Disease (COPD) represents one of the most significant public health challenges of the 21st century, constituting a leading cause of morbidity and mortality worldwide. According to the latest Global Burden of Disease studies, COPD affects approximately 212.3 million people globally, accounting for 3.3 million deaths annually and ranking as the fourth leading cause of death worldwide [1, 2]. The disease burden continues to escalate, with projections suggesting that global COPD prevalence will approach 600 million cases by 2050, representing a 23% relative growth compared to 2020 levels [3]. The World Health Organization defines COPD as a heterogeneous lung condition characterized by chronic respiratory symptoms including dyspnea, cough, expectoration and recurrent exacerbations due to abnormalities in the airways and alveoli that cause persistent, often progressive airflow obstruction [4]. This progressive nature of COPD, coupled with its irreversible pathophysiological changes, makes it a formidable clinical challenge requiring comprehensive understanding and management strategies. India bears a disproportionately high burden of COPD, contributing significantly to the global disease statistics. Current estimates suggest that India houses approximately 37.8 million COPD cases, representing 17.8% of the global COPD burden. More alarmingly, India accounts for a disproportionate 27.3% of global COPD deaths and 28.5% of global COPD disability-adjusted life years (DALYs), indicating substantial challenges in disease management and healthcare delivery [5]. Recent meta-analyses estimate the prevalence of COPD in India to be 7.4% among adults, with higher prevalence rates observed in urban areas (11%) compared to rural areas (5.6%) [6]. The disease burden varies significantly across different Indian states, with northern states including Uttar Pradesh, Bihar, West Bengal, and Rajasthan experiencing particularly high prevalence rates [5]. This geographical variation reflects the complex interplay of environmental, socioeconomic, and lifestyle factors that contribute to COPD development and progression in the Indian subcontinent. Tobacco smoking remains the most well-established and preventable risk factor for COPD development and progression. Scientific evidence consistently demonstrates that smoking is responsible for approximately 70-90% of COPD cases in high-income countries [7, 8]. The pathophysiological mechanisms underlying smoking-induced lung damage involve complex inflammatory processes, oxidative stress, and structural remodeling of airways and lung parenchyma. Cigarette smoke contains an extremely high concentration of reactive oxidants that induce inflammatory responses in the central airways, peripheral airways, and lung parenchyma. These inflammatory changes persist even in smokers with apparently normal lung function, suggesting that subclinical damage occurs long before clinical manifestations become apparent. The inflammatory process involves increased numbers of specific inflammatory cell types and structural remodeling resulting from repeated injury and repair cycles [9]. Spirometry remains the gold standard for COPD diagnosis and severity assessment, providing objective and reproducible measurements of airflow limitation [10, 11]. The Global Initiative for Chronic Obstructive Lung Disease (GOLD) criteria establish that a post-bronchodilator FEV_1_/FVC ratio of less than 0.70 confirms the presence of persistent airflow limitation and COPD diagnosis in patients with appropriate symptoms and significant exposure to noxious stimuli [4, 10]. The forced expiratory volume in one second (FEV_1_) serves as a crucial parameter for determining COPD severity, with GOLD classification stages ranging from mild (FEV_1_ ≥80% predicted) to very severe (FEV_1_ <30% predicted) [12, 13]. Beyond diagnosis, pulmonary function tests provide valuable insights into disease progression, treatment response, and prognosis. Studies have demonstrated that FEV_1_ decline rates vary across COPD severity stages, with accelerated decline often occurring in earlier disease stages [14]. The relationship between smoking exposure and pulmonary function decline represents a critical area of investigation in COPD research. Multiple studies have demonstrated that continued smoking significantly accelerates lung function deterioration in COPD patients. Research indicates that smoking cessation represents the most effective intervention for slowing disease progression and improving patient outcomes [15, 16]. Recent investigations have revealed complex relationships between smoking behavior patterns and pulmonary function outcomes. Early smoking initiation, particularly before adulthood, has been associated with more severe ventilatory dysfunction and accelerated disease progression. Studies comparing different smoking behaviors have shown that early-smoking groups demonstrate worse pulmonary function with significantly lower FEV_1_% predicted values and FEV_1_/FVC ratios compared to late-smoking groups [17]. Therefore, it is interest to examine smoking status and pulmonary function test parameters in Central Indian tertiary care COPD patients.

## Materials and Methods:

This study was a single-center, hospital-based, cross-sectional observational investigation conducted at the Department of Pulmonary Medicine, LN Medical College and JK Hospital, Bhopal, Central India. It aimed to explore the correlation between smoking history and pulmonary function test (spirometry) parameters among COPD patients.

## Study design and duration:

The study spanned 18 months, divided into three phases: planning (3 months), participant recruitment and data collection (12 months), and data analysis and report writing (3 months). Ethical clearance was obtained from the Institutional Ethics Committee of the hospital.

## Participants:

COPD patients aged 45 to 75 years, with an SPO2 level greater than 90%, were eligible for inclusion. Exclusion criteria included patients with an SPO2 level less than 90%, age below 45 or above 75, morbid obesity, unstable cardiac or cerebrovascular conditions, and those with other significant comorbidities like psychiatric illness or unstable COPD. Participants were recruited using a non-probability convenience sampling method from the hospital's outpatient registry, based on medical records and confirmed COPD diagnosis according to the GOLD guidelines.

## Data collection:

Upon obtaining informed consent, demographic data, smoking history (quantified in pack-years), and spirometry results (FEV_1_ and FVC) were collected. Spirometry was performed following the American Thoracic Society (ATS) and European Respiratory Society (ERS) guidelines, ensuring consistency and accuracy of the results. The best of three acceptable measurements for FEV_1_ and FVC was recorded.

## Primary and secondary outcomes:

The primary outcome was the correlation between smoking history and pulmonary function test results. The secondary outcome assessed the role of age and gender as potential confounding variables.

## Data handling and analysis:

Data were entered into a secure electronic database, with access restricted to authorized personnel. The dataset was cleaned and analyzed using Stata software (version 17.0). Descriptive statistics were used to summarize the data and correlation analysis and multivariable regression models were employed to assess the relationship between smoking history and lung function while adjusting for age and gender.

## Ethical considerations:

The study adhered to ethical standards ensuring patient confidentiality. Informed consent was obtained from all participants, and the study was conducted in line with institutional and national ethical guidelines.

## Funding and conflict of interest:

This study received no external funding, and the authors declare no conflict of interest. All expenses were covered by the study institute.

## Results:

Table 1 (see PDF) presents the gender distribution of COPD patients across different levels of pulmonary obstruction. The data reveals that males are overwhelmingly affected across all severities of COPD, with 80% of patients in the mild obstruction group being male, increasing to 100% in the very severe obstruction group. The percentage of females significantly drops as the severity of COPD increases, with only 20% in the mild obstruction group and no females in the very severe obstruction group. This indicates a male predominance in COPD severity (Table 1 - see PDF). Table 2 (see PDF) provides the distribution of participants based on their socioeconomic status (SES) by severity of COPD, classified using the Kuppuswamy SES scale. A higher percentage of participants in the severe and very severe obstruction categories belong to the upper middle class, with 45.1% in the severe group and 33.3% in the very severe group. The lower and upper lower classes are more prominent in the moderate and very severe groups, reflecting a potential socioeconomic gradient in the severity of COPD. Table 3 (see PDF) highlights the most common symptoms and signs experienced by patients across varying severities of COPD. Breathlessness was the predominant symptom across all groups, with its frequency increasing from the mild to very severe obstruction categories. Chest pain and productive cough were more common in the severe and very severe groups, with 33.3% of very severe COPD patients reporting chest pain. These findings emphasize the importance of breathlessness and chest pain in more severe stages of COPD (Table 3 - see PDF). Table 4 (see PDF) shows the correlation between the FEV_1_/FVC ratio and smoking-related variables. The results suggest a negative correlation between the FEV_1_/FVC ratio and both the duration of smoking (-0.46) and the smoking index (-0.59). Although the duration of smoking shows a marginally non-significant correlation with FEV_1_/FVC, the smoking index displays a stronger and statistically significant negative correlation (p = 0.041), indicating that higher tobacco exposure is associated with reduced pulmonary function. Table 5 (see PDF) focuses on the correlation between FEV_1_ (Forced Expiratory Volume in 1 second) and smoking-related variables. Both the duration of smoking (-0.61) and the smoking index (-0.68) show significant negative correlations with FEV_1_, with p-values of 0.023 and 0.017, respectively. These results suggest that longer smoking duration and higher smoking exposure are significantly associated with greater declines in FEV_1_, highlighting the detrimental impact of smoking on lung function (Table 5 - see PDF). Table 6 (see PDF) summarizes the general demographics and pulmonary function test (PFT) results of the study participants. The mean age of participants was 62 years, with a standard deviation of 9.36 years. Clinical parameters such as oxygen saturation, pulse rate, respiratory rate, systolic and diastolic blood pressures were also recorded, along with smoking-related measures such as the smoking index, pack year, and duration of smoking. This table provides an overview of the health status and smoking history of COPD patients, which correlates with their pulmonary function as detailed in the other tables.

## Discussion:

The present study elucidates key dimensions of the relationship between smoking history, pulmonary function, and COPD severity among patients in a tertiary care hospital in Central India. Consistent with national data, males predominated across all COPD severity categories, accounting for over 90% of moderate to very severe cases. This male predominance aligns with pooled prevalence estimates showing higher COPD rates in Indian men than women (11.4% vs. 7.4%) 13], and with an Indian hospital-based cohort where 70% of COPD patients were male against 30% female [18]. Gender-specific exposures-particularly higher rates of tobacco smoking in men-likely underpin this disparity, while lower smoking prevalence and potential hormonal protection in women may contribute to their underrepresentation in severe COPD groups. A study done by Rashmi *et al.* [19] reflects that there is high risk of COPD in smokers, with great concern for the mankind with the need to re-duce smoking. Contrary to many community studies where lower socioeconomic status (SES) correlates with more severe COPD, this study observed the greatest proportion of severe obstruction in upper-middle-class patients. Similar findings have emerged in hospital settings where direct and indirect costs of COPD management rose with airflow limitation severity, even in those with government subsidies [20], and where SES was not a major determinant of quality of life in tertiary-care COPD cohorts [21]. These discrepancies may reflect differential healthcare access, lifestyle exposures (e.g., indoor pollution, sedentary behaviors), and occupational hazards among higher-SES individuals who seek tertiary care. However, the presence of moderate and very severe COPD in lower-SES groups underscores persisting socioeconomic health disparities in COPD burden [22]. The study demonstrated a strong negative correlation between both duration of smoking and smoking index with FEV_1_/FVC ratio and FEV_1_% predicted. A similar inverse relationship between smoking index and vertebral bone density was reported (r = -0.627, p < 0.05), reflecting cumulative tobacco impact on pulmonary and extrapulmonary tissues [1]. Epidemiological surveillance has long established that higher smoking index predicts greater COPD incidence and more pronounced lung-function impairment (above 40% when smoking index is high) [23] and early-smoking groups exhibit worse FEV_1_% predicted and FEV_1_/FVC values than late initiators [24]. These findings reinforce the critical role of both intensity and duration of smoking in COPD progression. Dyspnea was the predominant symptom across all severity levels, intensifying with worsening obstruction. This mirrors clinical observations that females with COPD report dyspnea more frequently than males, despite lower smoking rates [18]. Productive cough and chest pain were mainly confined to severe and very severe stages, consistent with exercise-capacity studies in COPD smokers showing progressive symptom burden with declining lung function and reduced exercise tolerance [25]. The absence of productive sputum in mild cases suggests that mucus hypersecretion becomes clinically significant only with advanced airway remodeling and obstruction.

## Conclusion:

This study reveals significant insight into the demographics, smoking history, and clinical presentation of COPD patients in Central India. Thus, we show that there is significant impact of smoking on pulmonary function, with both the duration and intensity of smoking exacerbating the severity of the disease. Mitigating smoking as a primary risk factor along with improving early detection and intervention strategies will be essential in alleviating the burden of COPD in this area.
